# Physiotherapy Intervention in the Immediate Postoperative Phase of Lipedema Surgery—Observational Study

**DOI:** 10.3390/jcm14072137

**Published:** 2025-03-21

**Authors:** Ángela Río-González, Esther Delgado-Pérez, Elisa García-García, Laura González-Fernández, Sara García-Isidoro, Ester Cerezo-Téllez

**Affiliations:** 1 Asociación Española de Linfedema y Lipedema AEL, 28003 Madrid, Spain; angelariogon@gmail.com; 2 Department of Physiotherapy, Faculty of Medicine, Health and Sports, Universidad Europea de Madrid, 28670 Madrid, Spain; esther.delgado@universidadeuropea.es (E.D.-P.); elisa.garcia@universidadeuropea.es (E.G.-G.); laura.gonzalez@universidadeuropea.es (L.G.-F.); 3 Sanamanzana Physiotherapy Clinic, 28003 Madrid, Spain; 4 Woman & Health Research Group, Department of Physiotherapy, Faculty of Medicine, Health and Sports, Universidad Europea de Madrid, 28670 Madrid, Spain; saragey@gmail.com; 5 Faculty of Medicine and Health Sciences, Department of Nursing and Physiotherapy, Universidad de Alcalá, 28801 Madrid, Spain; 6 Neuromusculoskeletal Physiotherapy in Stages of Life Research Group (FINEMEV), Department of Nursing and Physiotherapy, Faculty of Medicine and Health Sciences, Universidad de Alcalá, Autovía A2, km 33.200, 28805 Madrid, Spain

**Keywords:** physiotherapy, liposuction, complications, treatment, pain, assessment

## Abstract

**Background**: Lipedema is an adipose tissue disorder in women, with an abnormal fat deposition in lower limbs and occasionally upper limbs. The condition is characterized by pain, bruising, heaviness, and mobility impairment. **Objectives**: This study aims to evaluate the effects of a modified Complete Decongestive Therapy protocol using the Godoy Method in the postoperative period following lipedema surgery. **Methods**: In total, 293 participants who underwent liposuction for lipedema were studied. The postoperative physiotherapy protocol included Godoy cervical stimuli, Manual Lymphatic Drainage based on Godoy maneuvers, mechanical lymphatic drainage with RAGodoy^®^, compression with bandages, skin care, and therapeutic education. **Results**: This study found that the number of physiotherapy sessions significantly reduced pain (*p* = 0.000) and other complications (*p* = 0.007) and increased mobility (*p* = 0.003). The number of physiotherapy sessions showed significant differences in pain intensity at 90 days post-treatment (*p* = 0.000). In total, 47.24% of the participants became functionally independent on the third day of the physiotherapy intervention (*p* = 0.003). A total of 40.96% of the participants developed some complications, although a relationship between inadequate compression and the occurrence of complications was also found in 36.52% of patients. **Conclusions**: The success of surgical treatment for lipedema not only depends on the surgery itself but also on the proper management of the patient in the perioperative period to minimize complications and prevent recurrence. The Complete Decongestive Therapy protocol modified with the Godoy Method showed effects on pain reduction, mobility increase, edema reabsorption, and prevention of complications, consequently enhancing functionality and quality of life for patients undergoing lipedema surgery.

## 1. Introduction

Lipedema, also known as lipohyperplasia dolorosa (LiDo), is an adipose tissue disorder, mostly affecting the lower limbs from the hip to the ankle, not affecting the feet, and sometimes affecting the arms, leaving the hand unaffected. Affecting primarily women, its etiology is not yet clearly defined [1]. The lack of differential diagnosis of lipedema, lipohypertrophy, lymphedema, and obesity make it difficult to establish a real supported prevalence. However, it is described between 5 and 19%. It is highly heterogeneous depending on the populations, locations, and countries in which the studies were performed, such as Germany [2,3], the United States [4], and Spain [5].

It is characterized by a disproportion between the lower limbs and the trunk. It is bilateral, symmetrical, chronic, painful, and does not ameliorate with weight loss or physical exercise [6,7]. Lipedema patients may experience pain to palpation, heaviness, ecchymosis, and bruising at minimal trauma; other signs include a negative Stemmer sign, cut-off or cuffing sign, sensitivity to cold, static edema, and telangiectasias [5,7,8,9]. Other connective tissue alterations may also appear, as well as misalignment of the lower limbs [10,11]. As aggravating factors, obesity, immobility, and venous or lymphatic insufficiency are described. Lipedema patients who are not overweight and exercise regularly have fewer symptoms and complications and better day-to-day functional performance [7,8,9].

Lipedema has a high biopsychosocial impact. It is sometimes associated with depression, anxiety, and eating disorders [12].

Treatment for lipedema can either be conservative or surgical [2,5,7,9,13,14]. The first is Complete Decongestive Therapy (CDT), which includes compression therapy, Manual Lymphatic Drainage (MLD), exercise, skin care, and hygienic-dietary measures [2,5,7,13]. CDT must be adapted to each patient. It can also reduce capillary fragility in lipedema patients [15]. MLD is effective in improving lipedema symptoms, reducing pain, discomfort, heaviness, and edema [7]. There are several international DLM schools, such as Vodder, Leduc, Foldi, etc., and the most recent is Godoy and Godoy, known since 1999. The Godoy Method is composed of four techniques or therapies that can be used in isolation (monotherapy) or together, increasing the effectiveness of the results in less time. These include Cervical Stimulation, Manual Lymphatic Drainage maneuvers, mechanical lymphatic drainage, and compression techniques. Compression garments support the tissues, help to reduce pain, shape the limbs, and, when associated with lymphatic or venous edema, the compression favors reabsorption [7,16].

A healthy lifestyle (diet and exercise) helps to maintain adequate weight, fewer symptoms and complications, and better functional capacity [8]. In cases where conservative treatment is not enough, the surgical option is another possibility [5]. This is indicated when the conservative approach fails because there are no satisfactory results, or a progression of symptoms occurs [2,4,13,17]. The failure of CDT applied for at least 6 months is used as a requirement for inclusion in the surgical process [14].

The main surgical intervention procedures currently used for lipedema involve tumescent liposuction, water-assisted liposuction, and lymphological liposculpture [7,17,18].

Liposuction requires the drainage of lymphatic fluid for several days after the procedure. The skin incisions may be open to ensure drainage of the wound’s secretion. Low-dose heparin and antibiotics are given after the procedure [19].

The conservative postoperative (PO) approach using MLD and compression is a well-proven procedure to prevent the accumulation of secretions, which can lead to complications [17]. Post-surgical care with CDT is advised by surgeons as part of the protocol and should be provided by a specialized physiotherapist as soon as possible to ameliorate edema, pain, and dysfunction [20].

In the early PO, MLD and compression should be considered as part of the therapeutic protocol [20,21], partly because of the need to rebalance lymphatic transport [17]. MLD was reported to not only reduce post-traumatic edema but to reduce levels of inflammatory mediators [22].

Different post-surgical conservative treatment protocols have been described. MLD and compression 2–3 times per week until the edema decreases have shown efficacy [23], while combining the treatment with other techniques like 24 h compression garments and physical activity [17], or the same for 4 to 5 weeks PO, has shown effectiveness [19]. The most used compression is nonelastic flat knitted or elastic circular knitted [7,24,25]. However, no gold standard has yet been described, so an application of CDT for lymphedema management in these patients is being used [4,7]. In addition, compression therapy recommendations may vary according to the patient’s choice, from 24 h compression garments for the first 7 days to 12 h overnight use only, to encourage patient adherence to treatment [17]. Depending on the patient’s situation, other compression protocols vary from 2 weeks to 2–3 months to prevent the accumulation of lymph in the treated areas and to control postoperative edema [19,23]. Occasionally, advanced lipedema or lymphatic complications may need compression for the rest of patients’ lives [18,23].

Modified CDT with intensive MLD sessions promotes decongestion and wound healing. Controlled therapeutic exercise, such as muscle activation pump, is essential for tissue decongestion. Bed rest is not indicated [17]. Additional complementary therapies such as acoustic wave therapy during 5 to 10 weeks [19] or mechanical lymphatic therapy RA Godoy^®^ [26,27] have shown decongestive effects in patients suffering from lymphatic problems. However, the protocol has not been described after the surgical procedure in lipedema patients.

Nowadays, as far as we know, the main PO complications described in lipedema patients are related to wound infections, seromas, necrosis, deep venous thrombosis, and erysipelas [2,17,28], which differ from those found in our clinical practice, so further studies addressing the immediate and subsequent PO phases are needed. Although these complications have already been described, further research is required to investigate the effects of physiotherapy on pain, mobility, and complications in post-surgery lipedema patients to develop more efficient and individualized treatment protocols.

We aim to gather data on the efficacy of a physiotherapy intervention with the modified Godoy Method in mitigating complications, such as fibrosis, seroma, and genital edema, as well as enhance skin health and resolve trophic disorders frequently encountered in our clinical experience.

## 2. Materials and Methods

### 2.1. Design

A retrospective cohort study was carried out in July 2024 with a sample treated from January 2019 to January 2023 in a private physiotherapy center in Madrid. The sample was composed of adult women referred from two private hospitals after having undergone the surgical proceeding. The patients remain in the hospital for at least 24 h to monitor possible major complications. After surgery, their surgeons recommended receiving CDT regardless of the operated body region and its previous state. All participants received the same physiotherapy treatment. The exclusion criteria were unilateral surgery, lack of data, or less than 2 sessions. This study was approved by the Clinical Research Committee of Hospital Universitario Principe de Asturias (CEIm OE36/2023).

### 2.2. Intervention and Assessment

Once the participant was discharged from the hospital, a complete physiotherapy assessment was performed. All sociodemographic data were recruited: month of intervention, body mass index (BMI), kind of surgery, extracted liters, psychological treatment, physiotherapy sessions, pain, mobility, complications (seroma, wound infection, chafing or risk of ulcer, pain, fibrosis, genital edema), compression, smoking, and satisfaction with the treatment received. For all participants, this was their first surgery. Three surgeons performed the surgical intervention using the same procedural method: the WAL technique.

The modified CDT physiotherapy protocol, based on Godoy’s Method, was applied to all study participants. The same defined protocol was applied to every participant. This protocol comprised 4 therapies of the Godoy Method cervical stimuli, manual and mechanical lymph drainage and compression, as detailed below: (1) Cervical stimuli—this is a maneuver performed at the base of the neck, with a frequency of 20–30 stimuli per minute for 15–20 min with the participant in a supine position. It is a superficial, rhythmic, painless maneuver in which the thumb pulls and slides from the area of the internal jugular vein towards the homolateral clavicle [27]. (2) MLD based on Godoy’s Method, using different manual maneuvers depending on the phase: maneuvers following lymphatic vessels with linear paths or intermittent compressions [29]. At the acute phase with recent wounds or scars, only intermittent compressions are used [29]. (3) Mechanical lymphatic drainage with RA Godoy^®^ device—passive ankle flexion and extension movements are performed for 50 min, at 20–25 cycles per minute [26]. (4) Compression therapy with multilayer and multicomponent bandages during the mechanical lymphatic drainage. (5) Skin care—before and after the bandages—and therapeutic education [2]. (6) Put on compression garments with the help of sliders. (7) Active movement (triple flexion, triple extension, and gait training) if possible.

### 2.3. Outcomes

The outcomes (pain, edema resorption, complications, mobility, and patient satisfaction) were collected before and after the physiotherapy intervention. Pain and patient satisfaction were also collected at the 90-day follow-up.

Both clinical and functional scales have been used to measure pain, mobility, and function related to real life over patients’ dependency.

The pain was measured by the validated 10-point visual analog scale (VAS) [30]. Patients were asked to describe their improvement in mobility and the reduction in bruising on a 3-point scale: mobility 1—dependent to move/no improvement, 2—somewhat dependent/minor to medium improvement, and 3—independent marked improvement [14]. Satisfaction with the treatment was measured by a verbal numeric scale from 0 to 10.

Edema resorption was made through standardized methods of graphic evaluation where different photos were taken using a defined protocol. This choice became necessary because of the impossibility of performing any centimetric measurement due to skin pain.

The number of sessions was first analyzed as a continuous outcome as clinical observation provides us with a perspective of the real effects of an isolated session, which are not significant. An analysis of the real effects of this approach had to be performed by using a batch of 5 sessions.

### 2.4. Statistical Analysis

A descriptive statistical analysis was performed using means and standard deviation for the quantitative variables and using percentages for dichotomous and categorical variables. An inferential analysis was carried out using paired and unpaired *t*-tests and one-way and repeated measures ANOVA to search for differences between quantitative variables. Pearson’s chi-square test was also used to relate dichotomous and categorical variables. All missing values were excluded from the analysis. In all cases, a *p*-value < 0.05 and a 95% confidence interval were used for the degree of statistical significance. Missing numbers were extracted from the analysis. All statistical analysis was performed using the Stata^®^ version 14.2 package for MS Windows^®^ version 10.

## 3. Results

**Figure 1:**
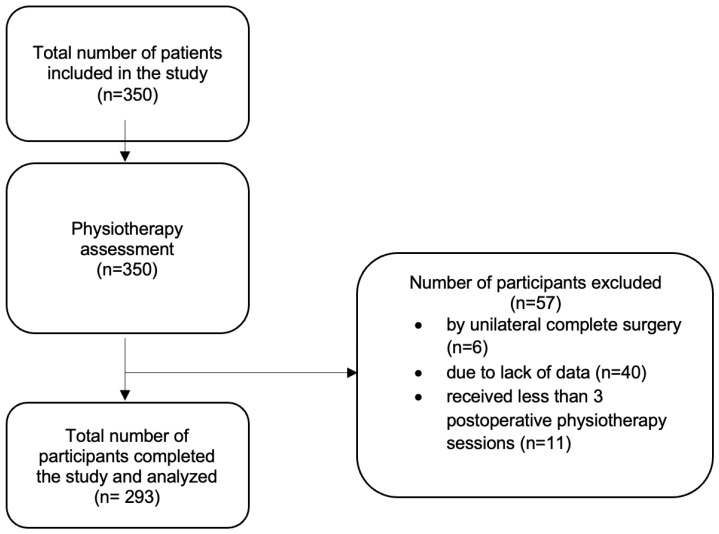
Study patient recruitment flowchart.

A total of 293 study participants were included in the analysis, and complete data were obtained for 97.6% of cases. The patient recruitment and follow-up process are shown in Figure 1. The sample was composed of women with a median age of 37,79. Most of the sample was overweight (42.31%), underwent surgery for lipedema in 2021 (40.27%), during the coldest months of the year (53.24%), and were operated on the calf area (47.60%). An average of 4.43 L was extracted during the surgery. The participants received an average of 6.68 physiotherapy sessions after the operation, and 47.24% of the participants were already independent on the third day after the physiotherapy intervention. In total, 40.96% of the participants developed some type of minor complication, and it was observed that the compression had not been adequate in 36.52% of the sample and that only 10.58% of the participants declared smoking (Table 1).

The number of physical therapy sessions was analyzed to determine whether they were related to the presence of complications, but no statistically significant differences were observed in any case (*p* < 0.05). Patients with general complications received a mean of 6.916 (0.350) physical therapy sessions, a similar number to the mean of 6.526 (0.295) sessions received by patients without complications.

Patients were categorized according to the number of physical therapy sessions they received after surgery: those who received fewer than 5 sessions, those who received 5 to 10 sessions, and those who received 10 or more sessions. The authors analyzed whether there were significant differences in the number of sessions received according to the complication experienced. It was observed that in the group that received less than 5 sessions, participants with fibrosis needed, on average, 0.446 more sessions than those who did not suffer from fibrosis (95% CI: 0.125–0.767) (*p* = 0.007) (Table 2).

When studying the effect of physiotherapy sessions on pain and mobility, statistically significant differences (*p* > 0.05) were seen between a high pain intensity before, after, and 90 days after treatment, as well as in mobility (Table 3).

**Figure 2:**
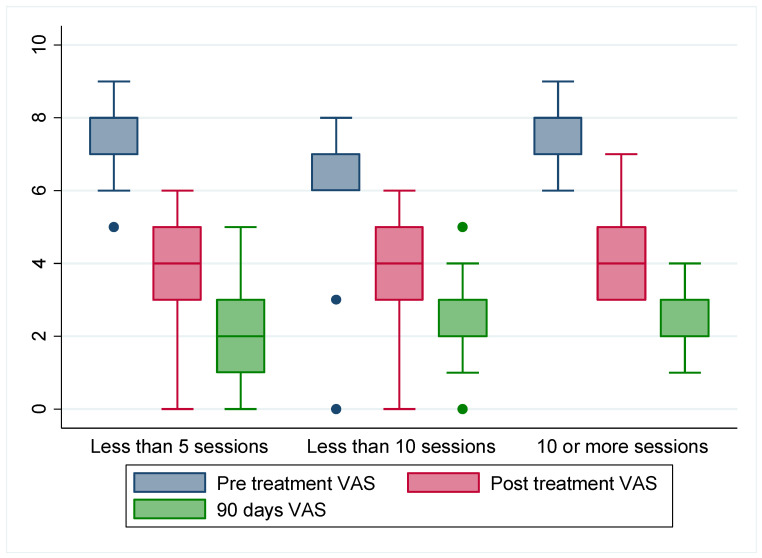
Distribution of VAS values according to groups of less than 5 sessions from 5 to 10 sessions and more than 10 physiotherapy sessions at different time lapses (pre-treatment, post-treatment, after 90 days). Colored dots represent isolated values of the sample.

The categorization of patients was carried out to investigate how the number of physiotherapy sessions affects pain. In all categories, significant differences were observed between the mean pain intensity of the different measurements, both when compared two to two (*p* = 0.000) and through repeated measures analysis (*p* = 0.000), which also yielded excellent goodness of fit (0.859 ≤ R2 ≥ 0.885). However, no differences were found between the before-treatment VAS values (*p* = 0.059) or after-treatment VAS values (*p* = 0.366) when testing the differences between the categories studied. Nonetheless, differences were found for 90 days after-treatment VAS values (*p* = 0.001), with a mean pain intensity in the “less than 5 sessions” group at 2.086 (0.094), the mean pain intensity in the “less than 10 sessions” group at 2.379 (0.095), and the mean pain intensity in the “more than 10 sessions” group at 2.667 (0.742). Interestingly, when studying the distribution of the sample, it was observed that despite the slightly higher pain intensity mean of the group that received 10 or more sessions, its minimum and maximum were much closer to the mean, resulting in more concentrated scores with lower values than the other groups for all patients (Figure 2). The more sessions that are carried out, the more accurately the results are expressed closer to the mean, confirming the benefits of the treatment regarding pain reduction. Furthermore, 100% of the participants referred to being proud of having been operated on, and edema reduced consistently in all of them.

## 4. Discussion

The aim of this study was to describe the effects of the modified CDT physiotherapy protocol, based on Godoy’s Method in decreasing pain, increasing mobility, edema resorption, and preventing surgical complications after liposuction in lipedema. All the participants were satisfied with the physiotherapy approach received. An interview was conducted during the physiotherapy consultation at the end of the last session. In general, after the failure of conservative treatment or worsening of symptoms, they decided to be operated on. However, they did not realize how painful or destabilizing the after-surgical proceeding was until they felt it themselves. Physiotherapy protocols help to recover in terms of mobility, pain, and consequently in function. Pain is the primary symptom and is a major cause of quality-of-life impairment [31].

The average pain intensity at 90 days was 2.341. Pain reduction is higher in our study in relation to other authors [14,23]. Dadras et al. [23] studied 25 patients and 72 liposuctions. The first postoperative follow-up was performed between 4 and 34 months after their last liposuction, with a reduction in VAS of 3.5 [14,23]. Wollina et al. [14] measured the pain in VAS before the first and the last liposuction. Their pain level reduced from 7.8 to 2.2 on 111 patients. There is no detailed post-surgery protocol, only related to wearing compression garments refitted when needed. Their patient’s liposuction was 4.7 L, and ours was 4.43 L. Furthermore, an amelioration of mobility and bruising is shown [14].

Cornelly et al. [17] reported 1400 surgeries but did not provide specific information on the quantification of pain. They report good results with a 4-week CDT program with 10 sessions and a similar reduction in complications as we found. We obtained a similar amelioration in 2 h treatment four or five times per week. Furthermore, the amelioration is maintained for a 90-day-long assessment. This might be due to the intensive number of hours and frequency of our physiotherapy treatment. We encourage the use of this intensive option as a treatment approach. In the first few hours, there is the most after-surgery edema, pain, hematoma, and ecchymosis. The synergic effects of the different therapies used with the modified CDT physiotherapy protocol, based on Godoy’s Method, favors a faster edema reabsorption, and thus a reduction in pain and bruising. Consequently, mobility is improved, increasing functionality. Despite not having collected outcomes about exercise in our studio, participants are advised to exercise as early as possible. Future lines of research should include these outcomes in the assessment.

According to the surgical technique used and the PO protocols, variations in complication records have been observed. In the present study, complications found (in specialized physiotherapy consultation) from 48 h after surgery and during the first 3 weeks have been considered. The most frequent complications found were seroma, pain, wound infection, genital edema, and chafing (friction wound, blister, and ulcer), appearing in 40.96% of participants. These complications have not been extensively described in previous studies or in the same acute period. As far as we know, in the most recent study [12] (retrospective), after 1400 lymphological liposculpture surgeries, complications were observed in 3.07%, including infections, seroma, erysipelas, necrosis, and deep venous thrombosis. In Kanapathy et al.’s metanalysis [28] of 23 articles involving 3583 patients, they reported an incidence of 11.62% minor surgical complications, with seroma being the most common. Other authors reported a complication rate of 9.5%, wound infections of 4.5%, and erysipelas of 4% [2]. The participants in our study did not present any erysipelas or deep venous thrombosis, perhaps because these are major complications, and the physiotherapy treatment could not be applied.

The low rate of complications seen in Cornelly et al.’s study [17] may also be due to the mandatory PO program over 4 weeks with modified CDT. They explain that fluid accumulation commonly happens in the lower legs, and when it is stagnant, it can cause bacterial growth and negative consequences like infections. Accentuated MLD and compression treatment can decrease the infection risk resulting from leg stasis. The high prevalence of complications shown in our study might be due to the inadequate compression observed in 36.52% of the sample. In the first days, a CDT approach is essential to prevent complications and improve pain. The indications should be individualized and surgical-area-dependent. Furthermore, the use of compression is essential, taking special care with surgery in the thigh area and the sanitary opening in compression garments due to the appearance of genital edema. The stimulation of the lymphatic system and its effects on the immune response favor edema and bruising reabsorption. However, after this surgical procedure, there is a risk of an inadequate compression related to the change in the volume of the limb. Based on our research findings, insufficient compression after surgery is associated with a significant risk of complications because of the lack of reabsorption or chafing. These complications include ulcers or chafing, infection, and genital oedema.

Through our findings, we confirm the importance of considering BMI as a risk of complication predictor despite not being the best indicator in patients with lipedema and interpreting these results with caution. The post-surgical treatment with modified CDT should be as intensive as possible, with daily sessions during the first two weeks based on our results, reaching up to four weeks, as shown by other authors [12,14]. The priority is (A) lymphatic drainage, (B) compression adapted (refitted when needed [24], or reinforced with circular garments above), (C) exhaustive monitoring of the skin, and (D) early supervised mobilization. Previous studies recommend wearing different types of compression [7,14,24], and like others, we think that the election of compression should be individualized [5,24,32].

Regarding the duration of PO, compressive therapy goes from a minimum of 2–4 weeks [19], to 2–3 months, or forever [18,23]. About 30% of patients stop wearing compression within 3 months after surgery [32]. In some cases, BMI may be a determining factor in maintaining compression due to the correlation between obesity and lymphatic disorders [33].

The medical teams in our Spanish sample recommend 8 weeks of flat knitted and reinforced compression garments 1 month after surgery until 3–6 months.

Surgical intervention positively influences volume, pain, heaviness, mobility, and quality of life. However, in accordance with Herbst et al.’s findings [32], our clinical observations reveal a gap in understanding the importance of adherence to a healthy lifestyle, particularly with diet and exercise, in maintaining medium- and long-term outcomes. This fact highlights the need for further research to validate measurement tools, data collection methods, and a universal classification system in clinical practice to minimize biases and improve accuracy.

Our results show the improvement obtained with the PO physiotherapy protocol and the relationship between the frequency and immediacy of treatment with the reduction in complications. Pain and mobility measurements before and after sessions, patient interviews, and the ability to put on compression garments and walk provide evidence of functional improvement.

One of the strengths of this study is that participants are assessed in a daily short-term period. This allows us to detect and treat possible complications immediately. Reliable, sensitive, and evidence-based physiotherapy techniques are used.

As the participants do not reside in the same location, the physiotherapy protocol could not be consistently implemented in terms of duration and frequency. Further, all the patients received exactly the same protocol of treatment, and all the sessions were registered. To avoid this limitation, and to be able to understand the results of the analysis, the data were stratified. The patients underwent surgery in private hospitals, paying for surgery and physiotherapy, indicating a predominantly medium–high socioeconomic level of the sample. Volume perimeter was not assessed through centimetric perimeter because of PO pain; however, some pictures were taken and compared to confirm the volume change.

Furthermore, individual factors such as car or air travel during the immediate postoperative period added to less physiotherapy sessions could potentially contribute to the observed complications. This manuscript confirms that participants receiving less than five PO physical therapy sessions presented a higher risk of developing complications. Moreover, participants presenting complications required more physical therapy sessions to solve them. The higher PO complications found among surgical procedures in Spain might be due to a less strict preoperative and PO protocol regarding compression and physical therapy recommendations. In addition, our sample was overweight or obese as an aggravating factor [7]. It should be accurately registered as well as diet, smoking, and other factors (psychological profile among others) with caution. No data from the follow-up of complications in the medium or long term were collected. We observed that the evolution of complications is faster and with less tissue suffering in patients who have made the intensive PO, so a randomized controlled trial should be made.

## 5. Conclusions

A modified CDT physiotherapy protocol based on Godoy’s Method showed itself to be effective in reducing pain, increasing mobility, favoring the reabsorption of edema, and preventing complications after lipedema surgery. This procedure shows a high satisfaction for the participants. The success of the surgical treatment for lipedema not only depends on the surgery itself but also on the proper management of the patient in the perioperative period to minimize complications and prevent recurrence. The more PO physiotherapy sessions that take place, the less risk of complications there is. Further research regarding decongestive physiotherapy and new approaches should be performed to confirm these results.

## Figures and Tables

**Table 1:** Baseline descriptive data of the studied sample.

Outcome Measure		N	$\bar{X}$	(SD)
Age		292	37.79	7.59
Extracted liters		293	4.43	1.33
Physiotherapy sessions per surgery		290	6.68	0.23
	Week 1		3.64	1.41
	Week 2		1.87	1.71
	Week 3		0.72	1.12
	Week 4		0.39	0.75
Pre-treatment VAS		293	7.04	0.92
Post-treatment VAS		293	3.98	0.95
90 days post-treatment VAS		293	2.34	0.95
**Outcome measure**		**N**	n	%
Body mass index	Normal weight	286	49	17.13
	Overweight		121	42.31
	Obesity		116	40.56
Year of intervention	2019	293	70	23.89
	2020		105	35.83
	2021		118	40.27
Hot month	No	293	156	53.24
	Yes		137	46.76
Kind of surgery	Calf	293	139	47.60
	Front thigh		73	25
	Hind thigh		28	9.59
	Arms		11	3.77
	Whole thigh		41	14.04
Psychological treatment		293	70	23.89
Mobility day 1	Independent	292	0	0
	Somewhat dependent		11	3.77
	Dependent		281	96.23
Mobility day 3	Independent	290	137	47.24
	Somewhat dependent		151	52.07
	Dependent		2	0.69
Complications		293	120	40.96
	Seroma		60	20.48
	Wound infection		33	11.26
	Chafing or risk of ulcer		55	18.77
	Persistent pain		51	17.41
	Asymmetrical pain		36	12.29
	Fibrosis		155	52.9
	Genital edema		43	14.68
Smoking		293	31	10.58
Inadequate compression		293	107	36.52

$\bar{X}$: median; SD: standard deviation; *n*: sample; N: total sample; VAS: visual analog scale.

**Table 2:** Differences in the number of physiotherapy sessions according to the different complications analyzed, categorized according to the number of sessions.

		Less Than 5 Sessions			Less Than 10 Sessions			More Than 10 Sessions		
		$\bar{X}$ (SD)	Mean Difference (95% CI)	*p*-Value	$\bar{X}$ (SD)	Mean Difference (95% CI)	*p*	$\bar{X}$ (SD)	Mean Difference (95% CI)	*p*-Value
Complications	Yes	3.152 (0.788)	−0.108 (−0.445–0.229)	0.526	6.789 (1.277)	−0.509 (−1.040–0.023)	0.060	12 (1.515)	−0.0217 (−0.833–0.790)	0.957
	No	3.044 (0.953)			6.28 (1.278)			11.978 (2.016)		
Seroma	Yes	3.292 (0.888)	−0.258 (−0.661–0.145)	0.207	6.947 (1.268)	−0.579 (−1.232–0.073)	0.081	12.118(1.654)	−0.165 (−1.151–0.822)	0.740
	No	3.033 (0.954)			6.368 (1.284)			11.953 (1.855)		
Wound infection	Yes	3.333 (0.779)	−0.275 (8–0.8115–0.2629)	0.313	6.7 (1.338)	−0.241 (−1.104–0.622)	0.580	11.546 (1.573)	0.512 (−0.656–1.680)	0.385
	No	3.059 (80.8999)			6.459 (1.296)			12.057 (1.841)		
Chafing or risk of ulcer	Yes	3.227 (0.685)	−0.173 (−0.591–0.245)	0.414	6.625 (5.983)	−0.169 (−0.877–0.539)	0.636	12.125(1.708)	−0.1715 (−1.180–0.838)	0.736
	No	3.054 (0.930)			6.456 (1.318)			11.954 (1.841)		
Persistent pain	Yes	3.235 (0.903)	−0.173 (−0.637–0.290)	0.460	6.714 (1.271)	−0.295 (−0.932–0.341)	0.359	12.667(1.723)	−0.797 (−1.915–0.320)	0.159
	No	3.062 (0.888)			6.419 (1.303)			11.870 (1.806)		
Asymmetrical pain	Yes	3.25 (0.888)	−0.181 (−0.720–0.357)	0.506	6.273 (1.272)	0.239 (−0.589–1.066)	0.567	12.769(1.589)	−0.931 (−2.006–0.144)	0.088
	No	3.069 (0.893)			6.512 (1.303)			11.838(1.817)		
Fibrosis	Yes	3.295 (0.823)	−0.446 (−0.767–0.125)	0.007	6.546 (1.284)	−0.114 (−0.646–0.417)	0.671	11.939(1.463)	0.124 (−0.698– 0.945)	0.765
	No	2.849 (0.907)			6.431 (1.315)			12.063(2.257)		
Genital edema	Yes	3.235 (0.903)	−0.173 (−0.637–0.290)	0.460	6.667 (1.397)	−0.217 (−0.943–0.510)	0.555	11.272(1.272)	0.827 (−0.331–1.986)	0.159
	No	3.062(0.888)			6.45 (1.282)			12.1 (1.858)		

SD: standard deviation; CI: confidence interval; $\bar{X}$: median; *p*: *p*-value.

**Table 3:** Effect of physiotherapy on pain intensity and mobility.

	$\mathrm{Pain}\;\bar{X}$ (SD)				Mobility N					
		Pre-treatment VAS	Post-treatment VAS	90 Days Post-Treatment VAS			Mobility Day 3			
	$\bar{X}$ (SD)	7.041 (0.917)	3.976 (0.984)	2.341 (0.947)			Independent	Somewhat Dependent	Dependent	Total
Pre-treatment VAS	Mean difference (95% CI)		3.065 (2.911–3.219)	4.700 (4.548–4.851)	Mobility day 1	Independent	0	0	0	0
	*p*		0.000	0.000						
Post-treatment VAS	Mean difference (95% CI)			1.635 (1.478–1.792)		Somewhat dependent	10	0	0	10
	*p*			0.000						
90 days post-treatment VAS	Mean difference (95% CI)					Dependent	127	151	2	280
	*p*					Total	137	151	2	290
						χ^2^ (2) = 11.567		*p* = 0.003		

SD: standard deviation; VAS: visual analog scale; CI: confidence interval; $\bar{X}$: median; *p*: *p*-value; N: sample.

## Data Availability

Data are unavailable due to privacy or ethical restrictions.

## Abbreviations

LiDo	Lipohyperplasia dolorosa
CDT	Complete Decongestive Therapy
MLD	Manual Lymphatic Drainage
PO	Postoperative
BMI	Body mass index
VAS	Visual Analogue Scale
